# Rethinking miRNAs in MSC-sEV therapeutics: implications for manufacture, mechanism of action, and development of robust potency CQAs

**DOI:** 10.20517/evcna.2025.55

**Published:** 2025-11-19

**Authors:** Thong Teck Tan, Sai Kiang Lim

**Affiliations:** ^1^Paracrine Therapeutics Pte. Ltd., Singapore 536464, Singapore.; ^2^Department of Surgery, Yong Loo Lin School of Medicine, National University of Singapore, Singapore 119228, Singapore.

**Keywords:** Mesenchymal stem/stromal cell (MSC), small extracellular vesicle (sEV), miRNA, critical quality attribute (CQA)

## Abstract

Mesenchymal stromal cell-derived small extracellular vesicles (MSC-sEVs) have emerged as a promising cell-free alternative to MSC-based therapies, offering superior safety, scalability, and stability profiles. These nanosized vesicles are now widely regarded as the principal therapeutic effectors of MSCs, capable of recapitulating many of the benefits attributed to their parental cells. However, their successful clinical translation depends on overcoming key challenges, particularly those related to product variability, viral safety, and the definition of mechanistically relevant potency-associated critical quality attributes (CQAs). This review explores the sources of MSC-sEV variability, including MSC tissue origin, manufacturing parameters, and limitations associated with primary and pluripotent stem cell-derived MSCs. The use of immortalized monoclonal MSC lines is discussed as a potential solution to improve batch consistency. Regulatory frameworks such as the International Council for Harmonisation (ICH) guideline Q5A(R2) are also highlighted for ensuring viral safety in sEV manufacturing processes. A major focus is the critical evaluation of microRNAs (miRNAs) - long regarded as leading candidates for potency CQAs in MSC-sEV products. Despite their prevalence in the extracellular vesicle literature, mounting evidence challenges their functional relevance in therapeutic contexts. Studies consistently show that miRNAs are underrepresented in sEVs, occur at very low copy numbers, and lack essential components (e.g., Argonaute proteins) required for canonical RNA interference. Moreover, the efficiency of EV internalization and endosomal escape remains exceedingly low, rendering miRNA-based gene regulation mechanistically implausible at physiologically relevant doses. These findings call into question the widespread assumption that miRNAs are primary effectors of MSC-sEV activity.

## INTRODUCTION: FROM CELLS TO VESICLES - RETHINKING MSC THERAPEUTIC ACTIVITY

The discovery of mesenchymal stromal cell-derived small extracellular vesicles (MSC-sEVs) has fundamentally reshaped our understanding of MSC-based therapies. Initial studies revealed that the therapeutic benefits of MSCs were not dependent on their long-term engraftment or differentiation. A seminal 2002 review by Chopp and Li highlighted that both implanted and intravenously administered MSCs yielded similar functional recovery despite minimal *in vivo* persistence or differentiation^[1]^. These findings led to the hypothesis that MSCs mediate tissue repair predominantly through the secretion of bioactive factors - such as granulocyte colony-stimulating factor (G-CSF), stem cell factor (SCF), leukemia inhibitory factor (LIF), macrophage colony-stimulating factor (M-CSF), interleukin (IL)-6, and IL-11^[2,3]^.

While initial efforts focused on soluble proteins and small molecules, a pivotal 2008 study revealed that the active fraction of the MSC secretome exceeded 1,000 kDa, suggesting the involvement of large macromolecular complexes^[4]^. In 2009, Bruno *et al*. reported that MSC-derived microvesicles (80-1,000 nm) could ameliorate acute kidney injury in mice^[5]^. Shortly thereafter, Lai *et al*. demonstrated that exosomes (~100-130 nm) - a subtype of small extracellular vesicles - conferred therapeutic benefit in a myocardial ischemia model^[6]^. In follow-up work, Bruno *et al*. further refined the active vesicle size to < 200 nm^[7]^.

These insights catalyzed a paradigm shift, establishing MSC-sEVs within the 100-200 nm range - including exosomes and small microvesicles - as the principal effectors of MSC function^[8]^. Numerous preclinical studies have shown that MSC-sEVs can recapitulate or even exceed the therapeutic efficacy of their parental MSCs across diverse disease models^[5,9-11]^, positioning them as a promising class of next-generation, cell-free regenerative therapeutics.

## ADVANTAGES OVER CELL-BASED THERAPIES

MSC-sEVs offer several distinct advantages over conventional cell-based therapies. As non-living, non-replicative biological entities, MSC-sEVs inherently avoid risks associated with viable cell administration, including tumorigenicity, ectopic tissue formation, and uncontrolled proliferation. Their nanoscale size minimizes the risk of vascular occlusion following systemic delivery and enables sterile filtration - an essential feature for meeting Good Manufacturing Practice (GMP) requirements in scalable, standardized bioproduction.

A major logistical advantage of MSC-sEVs lies in their stability. They can be lyophilized without compromising structural integrity or functional potency, facilitating long-term storage at ambient or refrigerated conditions. This dramatically reduces reliance on cold chain logistics, one of the major cost and infrastructure barriers limiting the widespread deployment of cell therapies. Such shelf stability supports broader geographic access, point-of-care administration, and the development of off-the-shelf formulations for both topical and parenteral applications.

**Figure 1:**
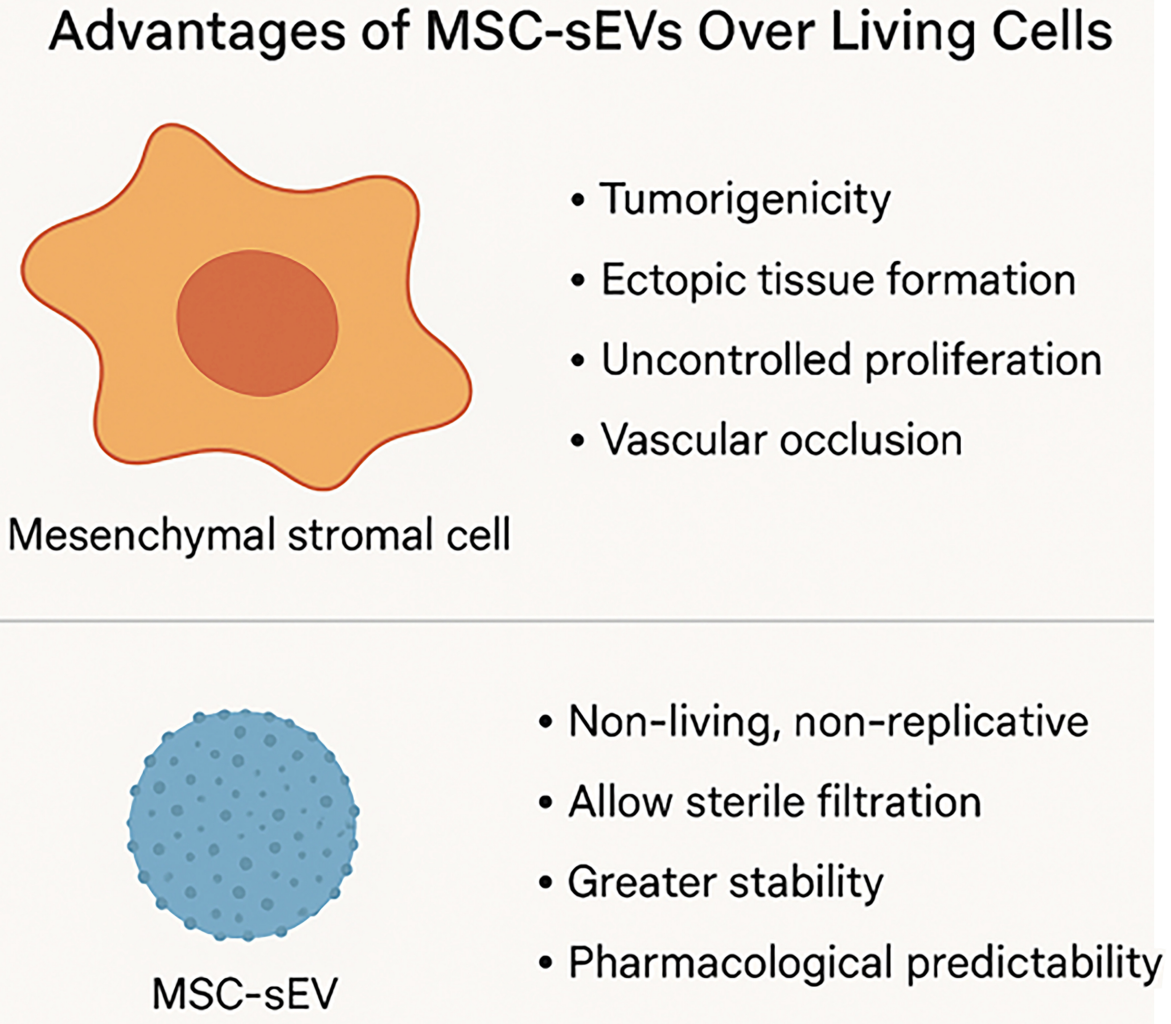
Diagrammatic summary: advantages of MSC-sEVs over MSCs (Figure was created with AI-assisted technologies). MSC: Mesenchymal stem cell; sEV: small extracellular vesicle.

Importantly, MSC-sEVs also exhibit greater pharmacological predictability. Unlike living MSCs - which dynamically respond to complex and often unpredictable *in vivo* cues - MSC-sEVs deliver a more consistent therapeutic profile, reflective of their defined *in vitro* characteristics. This reduced biological variability enhances their reproducibility in preclinical and clinical settings and facilitates more straightforward potency testing and regulatory evaluation [Figure 1].

Taken together, these properties position MSC-sEVs as a practical, safe, and scalable alternative to living cell therapies across a wide range of clinical and therapeutic applications. Notably, MSC-sEVs have already entered clinical evaluation, marking a critical step toward their therapeutic translation [Table 1].

**Table 1:** A list of MSC EV-based clinical trials registered at clinicaltrials.gov [Search terms: Intervention AND MSC AND (exosomes OR extracellular vesicles; date 19 August 2025)]

**NCT number**	**Study title**	**Study URL**
NCT02138331	Effect of microvesicles and exosomes therapy on Î^2^-cell Mass in T1DM	https://clinicaltrials.gov/study/NCT02138331
NCT03384433	Allogenic mesenchymal stem cell derived exosome in patients with acute ischemic stroke	https://clinicaltrials.gov/study/NCT03384433
NCT03437759	MSC-exos promote healing of macular holes	https://clinicaltrials.gov/study/NCT03437759
NCT04173650	MSC EVs in dystrophic epidermolysis bullosa	https://clinicaltrials.gov/study/NCT04173650
NCT04356300	Exosome of mesenchymal stem cells for multiple organ dysfuntion syndrome after surgical repaire of acute type a aortic dissection [Note: The title contains spelling errors (“Dysfuntion” and “Repaire”) in the original publication.]	https://clinicaltrials.gov/study/NCT04356300
NCT04366063	Mesenchymal stem cell therapy for SARS-CoV-2-related acute respiratory distress syndrome	https://clinicaltrials.gov/study/NCT04366063
NCT04388982	The safety and the efficacy evaluation of allogenic adipose MSC-exos in patients with alzheimer’s disease	https://clinicaltrials.gov/study/NCT04388982
NCT04491240	Evaluation of safety and efficiency of method of exosome inhalation in SARS-CoV-2 associated pneumonia	https://clinicaltrials.gov/study/NCT04491240
NCT04602442	Safety and efficiency of method of exosome inhalation in COVID-19 associated pneumonia	https://clinicaltrials.gov/study/NCT04602442
NCT04798716	The use of exosomes for the treatment of acute respiratory distress syndrome or novel coronavirus pneumonia caused by COVID-19	https://clinicaltrials.gov/study/NCT04798716
NCT04850469	Study of MSC-exo on the therapy for intensively ill children	https://clinicaltrials.gov/study/NCT04850469
NCT05060107	Intra-articular injection of MSC-derived exosomes in knee osteoarthritis (ExoOA-1)	https://clinicaltrials.gov/study/NCT05060107
NCT05216562	Efficacy and safety of EXOSOME-MSC therapy to reduce hyper-inflammation in moderate COVID-19 patients	https://clinicaltrials.gov/study/NCT05216562
NCT05243368	Evaluation of personalized nutritional intervention on wound healing of cutaneous ulcers in diabetics	https://clinicaltrials.gov/study/NCT05243368
NCT05261360	Clinical efficacy of exosome in degenerative meniscal injury	https://clinicaltrials.gov/study/NCT05261360
NCT05402748	Safety and efficacy of injection of human placenta mesenchymal stem cells derived exosomes for treatment of complex anal fistula	https://clinicaltrials.gov/study/NCT05402748
NCT05499156	Safety of injection of placental mesenchymal stem cell derived exosomes for treatment of resistant perianal fistula in crohn’s patients	https://clinicaltrials.gov/study/NCT05499156
NCT05523011	Safety and tolerability study of MSC exosome ointment	https://clinicaltrials.gov/study/NCT05523011
NCT05669144	Co-transplantation of mesenchymal stem cell derived exosomes and autologous mitochondria for patients candidate for CABG surgery	https://clinicaltrials.gov/study/NCT05669144
NCT05738629	Safety and efficacy of pluripotent stem cell-derived mesenchymal stem cell exosome (PSC-MSC-Exo) eye drops treatment for dry eye diseases post refractive surgery and associated with blepharospasm	https://clinicaltrials.gov/study/NCT05738629
NCT05808400	Safety and efficacy of umbilical cord mesenchymal stem cell exosomes in treating chronic cough after COVID-19	https://clinicaltrials.gov/study/NCT05808400
NCT05808400	Safety and efficacy of umbilical cord mesenchymal stem cell exosomes in treating chronic cough after COVID-19	https://clinicaltrials.gov/study/NCT05808400
NCT05871463	Effect of mesenchymal stem cells-derived exosomes in decompensated liver cirrhosis	https://clinicaltrials.gov/study/NCT05871463
NCT05940610	The safety and efficacy of MSC-EVs in acute/acute-on-chronic liver failure	https://clinicaltrials.gov/study/NCT05940610
NCT06202547	Intra-ovarian injection of MSC-EVs in idiopathic premature ovarian failure	https://clinicaltrials.gov/study/NCT06202547
NCT06279741	Safety and efficacy of MSC-EVs in the prevention of BPD in extremely preterm infants	https://clinicaltrials.gov/study/NCT06279741
NCT06431152	Intra-articular injection of UC-MSC exosome in knee osteoarthritis	https://clinicaltrials.gov/study/NCT06431152
NCT06545175	Intracochlear application of VSF1.01 for the reduction of cochlear implant surgery related trauma	https://clinicaltrials.gov/study/NCT06545175
NCT06598202	Exploring nasal drop therapy with small extracellular vesicles for ALS	https://clinicaltrials.gov/study/NCT06598202
NCT06599346	Effects of mesenchymal stem cell supernatant on prevention and treatment of skin/mucosal injury in hematology patients	https://clinicaltrials.gov/study/NCT06599346
NCT06812637	Efficacy and safety of wharton’s jelly-derived mesenchymal stem cell exosomes in the treatment of diabetic foot ulcers: a double-blinded randomized controlled clinical trial	https://clinicaltrials.gov/study/NCT06812637
NCT06825572	MSC-EVs in acute/ acute-on-chronic liver failure after liver transplantation	https://clinicaltrials.gov/study/NCT06825572
NCT06866184	Regenerative effects of birth material derived extracellular vesicles	https://clinicaltrials.gov/study/NCT06866184
NCT06919380	Nebulized MSC-exos for Anti-MDA5+ RP-ILD: safety and efficacy trial	https://clinicaltrials.gov/study/NCT06919380
NCT06995625	STEVIA	https://clinicaltrials.gov/study/NCT06995625

MSC: Mesenchymal stem cell; EVs: extracellular vesicles; Exo/Exos: exosome/exosomes; PSC: pluripotent stem cell; UC-MSC: umbilical cord mesenchymal stem cell; BPD: bronchopulmonary dysplasia; CABG: coronary artery bypass grafting; ALS: amyotrophic lateral sclerosis; RP-ILD: rapidly progressive interstitial lung disease; MDA5: melanoma differentiation-associated gene 5; STEVIA: STem cell-derived extracellular vesicle therapy in acute ischemic stroke; T1DM: type i diabetes mellitus.

## SOURCES OF VARIABILITY IN MSC-sEV PRODUCTS

### Manufacturing variables

The composition and functional attributes of MSC-sEVs are highly sensitive to upstream manufacturing conditions^[12,13]^. Key sources of variability include:

· The tissue origin of MSCs [e.g., bone marrow^[14]^, adipose tissue^[15]^, placenta^[16]^, umbilical cord^[17]^, embryonic^[18]^ or induced pluripotent stem cells (PSCs)^[19]^]; · Culture format (2D monolayer *vs.* 3D aggregates or bioreactors); · Medium supplements (e.g., fetal bovine serum, human platelet lysate); · Isolation and enrichment methods (e.g., ultracentrifugation, size-exclusion chromatography, tangential flow filtration).

Among these, the MSC tissue source is especially impactful, as it influences the baseline transcriptomic, proteomic, and secretory profiles of both cells and their derived sEVs.

### Limitations of primary and PSC-derived MSCs

While primary MSCs are widely used and supported by existing clinical experience, they face inherent limitations such as donor-to-donor heterogeneity, finite expansion potential, and senescence-associated functional decline^[12,13]^. Similar challenges affect MSCs derived from PSCs, which - although scalable - may still exhibit batch-to-batch variability and developmental heterogeneity^[18]^.

### Impact of cellular heterogeneity on sEV consistency

**Figure 2:**
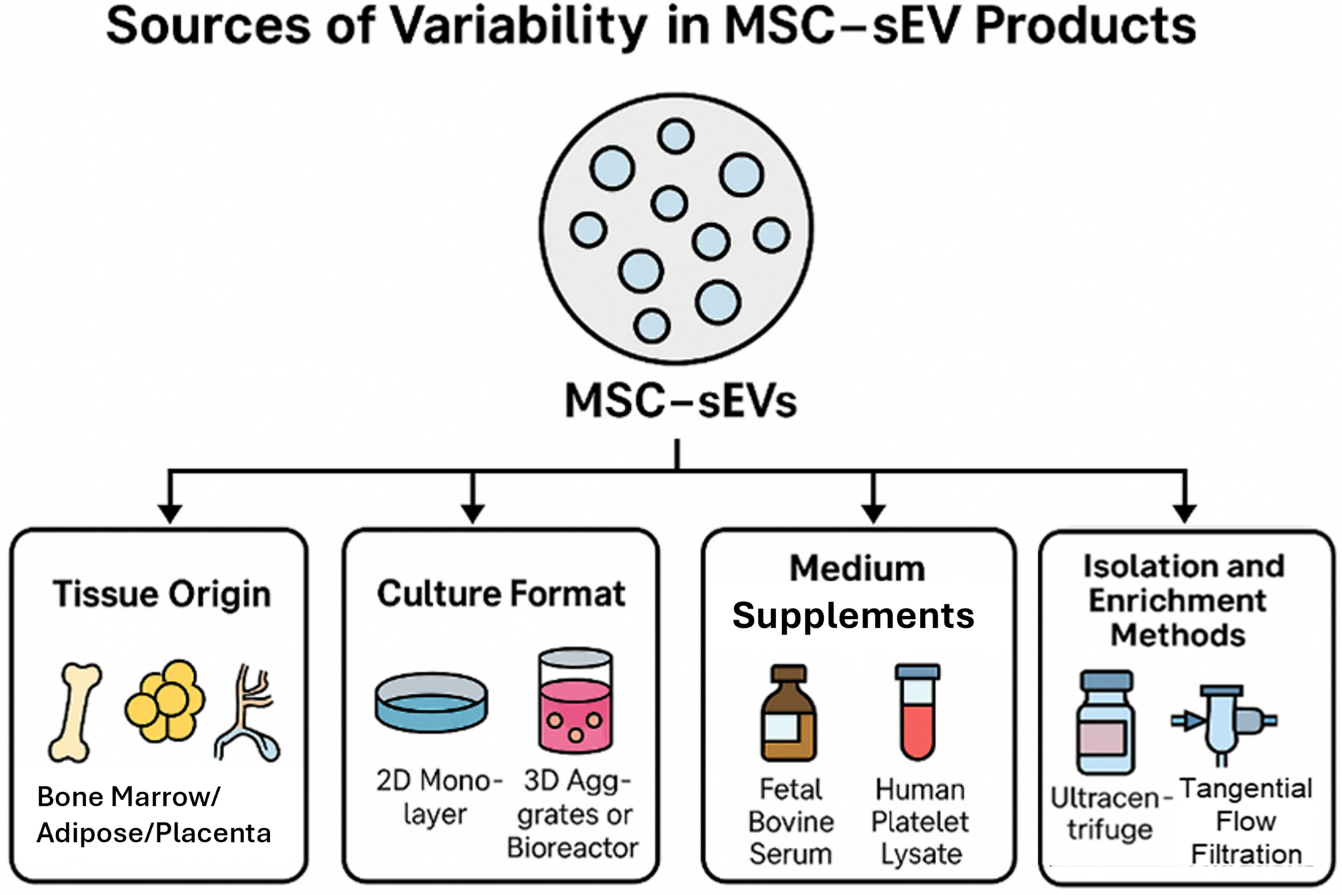
An overview of the key variables in the manufacturing of MSC-sEVs. (Figure was created with AI-assisted technologies). MSC: Mesenchymal stem cell; sEV: small extracellular vesicle.

A critical but unresolved issue is how variability at the cellular level translates to variability in sEV products. Although some early studies suggested general functional conservation among MSC-sEVs^[20]^, more rigorous comparative studies contradict this view. For example, Madel *et al*. (2023) demonstrated significant differences in immunomodulatory activity among sEVs derived from MSCs of different donors - even when cultured and processed under identical conditions^[21]^. These findings emphasize that both intrinsic factors (e.g., donor genetics, cell age) and extrinsic factors (e.g., media, isolation method) contribute to variability in MSC-sEV profiles [Figure 2].

## TOWARD CONSISTENT AND SAFE MSC-sEV PRODUCTION

### Immortalized monoclonal MSC lines: a path to standardization

GMP compliance is necessary but insufficient to eliminate biological variability. GMP addresses process control and documentation but does not solve inherent issues related to donor sourcing, cellular heterogeneity, or replicative senescence^[22,23]^. Thus, even under GMP, consistent MSC-sEV production remains elusive unless variability at the source cell level is addressed. To mitigate these issues, Chen *et al*. proposed the use of immortalized, clonally selected MSC lines and this was subsequently adopted by others^[24,25,26]^. These cell lines offer theoretically unlimited expansion potential and allow for tighter control over input variability. When derived from a single MSC clone and appropriately characterized, such lines provide a more reproducible and scalable source of sEVs. Despite these advantages, immortalized monoclonal MSCs are not exempt from genetic and epigenetic drift. Asymmetric division, spontaneous mutations, or subtle transcriptomic shifts can occur over extended passaging as widely documented in other cell lines^[13,21]^. In the context of MSC-derived extracellular vesicles (MSC-EVs), passage number - and by extension, the cumulative impact of cellular drift - has been shown to affect both the potency and yield of secreted vesicles^[27-29]^. Consequently, rigorous clone selection, passage number control, and routine molecular fingerprinting are essential to preserve product consistency. Functional testing, including proteomic and RNA profiling of sEVs, remains critical, reinforcing the principle that “the process defines the product”.

### Mitigating viral risk

The nanoscale dimensions of sEVs - typically 50 to 200 nm - place them in the same size range as many viruses, making viral safety a critical concern in MSC-sEV manufacturing. Unfortunately, conventional viral inactivation and removal methods - such as low pH exposure, heat treatment, and nanofiltration - are either ineffective or incompatible with sEVs. These procedures often disrupt membrane integrity, compromise bioactivity, or result in significant product loss. Given these limitations, compliance with International Council for Harmonisation (ICH) guideline Q5A(R2) (https://database.ich.org/sites/default/files/ICH_E6%28R3%29_DraftGuideline_2023_0519.pdf) is essential for ensuring viral safety in MSC-sEV products. This international guideline - titled “*Viral Safety Evaluation of Biotechnology Products Derived From Cell Lines of Human or Animal Origin*” - provides a harmonized framework for the detection, control, and risk mitigation of viral contaminants in biologics.

Under ICH Q5A(R2), manufacturers are required to:

· Characterize and qualify all source cell lines, including documentation of origin, history, and susceptibility to viral infection; · Conduct extensive adventitious agent testing of cell banks and raw materials, using both *in vitro* and *in vivo* assays as appropriate; · Implement rigorous aseptic processing controls, including closed systems and sterile filtration (where compatible); · Evaluate the overall viral clearance capacity of the manufacturing process, even if traditional clearance steps (e.g., solvent/detergent treatment, chromatography) are inapplicable to sEVs.

For MSC-sEV platforms, adherence to ICH Q5A(R2) ensures that viral risk is mitigated through upstream controls, rather than relying on downstream inactivation. This places greater emphasis on:

· The use of thoroughly screened and qualified master cell banks; · Serum-free or xeno-free media components; · Aseptic bioreactor systems with validated cleaning procedures.

**Figure 3:**
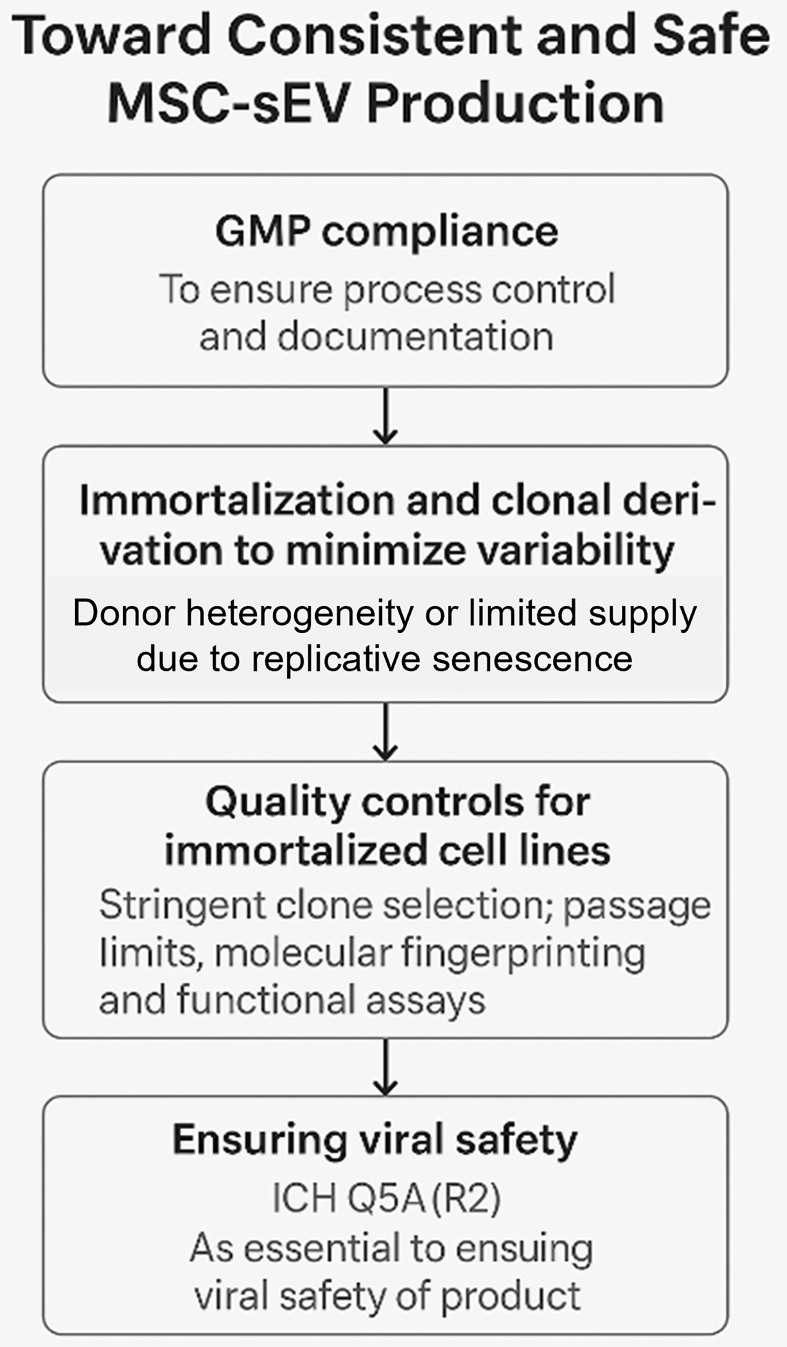
A critical path to consistent and safe MSC-sEV production. (Figure was created with AI-assisted technologies). MSC: Mesenchymal stem cell; sEV: small extracellular vesicle; GMP: Good Manufacturing Practice; ICH: International Council for Harmonisation of Technical Requirements for Pharmaceuticals for Human Use.

In summary, ICH Q5A(R2) provides the regulatory foundation for addressing the unique viral safety challenges of MSC-sEVs, especially when conventional purification techniques are not viable. Early integration of these principles into process development is essential for regulatory approval and safe clinical translation [Figure 3].

## THE NEED FOR FUNCTIONAL POTENCY CRITICAL QUALITY ATTRIBUTES

Establishing functional potency critical quality attributes (CQAs) is essential for the development, manufacturing, and regulatory evaluation of MSC-sEV therapeutics. Potency CQAs serve as proxies for the biological activity of the final product, providing assurance of consistent efficacy across batches and aligning with regulatory expectations for biologics. According to the consensus roadmap published by SOCRATES, ISEV, and ISCT^[30]^, the identification of potency CQAs must be rooted in a mechanistic understanding of how sEVs mediate therapeutic effects *in vivo*.

Traditionally, potency has been inferred from the molecular content of EVs - particularly microRNAs (miRNAs) - assumed to regulate gene expression in recipient cells through canonical RNA interference (RNAi) pathways. However, recent insights from pharmacokinetics, biodistribution, and mechanistic studies challenge this assumption. A comprehensive re-evaluation of the RNA-centric view of sEV function is warranted, particularly in light of the inefficiencies in EV uptake, endosomal escape, and the minimal stoichiometry of RNA cargo. This chapter outlines the current limitations of miRNAs as potency CQAs and highlights the need to reorient potency metrics toward more plausible mechanisms such as extracellular or receptor-mediated pathways.

### Rethinking the miRNA-centric mechanism of action

#### The cargo delivery paradigm: a failing model

The canonical model suggests that MSC-sEVs deliver functional cargo - particularly miRNAs - into the cytoplasm of recipient cells to mediate therapeutic effects. However, recent *in vivo* studies using radiolabeled, fluorescently tagged, or barcoded EVs consistently show that fewer than 1% of intravenously administered EVs are internalized by target cells^[31,32]^. Moreover, within this internalized fraction, the vast majority of EVs are trafficked to lysosomes, where cargo is degraded, with minimal evidence of cytosolic release^[33]^.

This internalization inefficiency presents a major barrier for functional RNA delivery. Engineering efforts to improve cellular uptake and endosomal escape, such as the incorporation of fusogenic proteins including VSV-G (vesicular stomatitis virus glycoprotein), have enhanced the functional delivery of catalytic proteins such as CRE recombinase into cells^[34]^. However, there is little evidence that VSV-G could facilitate delivery of miRNA to achieve functionally relevant miRNA concentrations in recipient cells at clinically feasible doses.

#### Extracellular mechanisms

The growing skepticism around the intracellular delivery hypothesis has prompted increasing attention to extracellular mechanisms of action as more biologically plausible alternatives. Rather than viewing EVs solely as cargo shuttles, these emerging models emphasize the biophysical and biochemical properties of EVs as active participants in cell communication.

One such model is the Vesicle-Induced Receptor Sequestration (VIRS) hypothesis, proposed by Jahnke and Staufer^[35]^. VIRS represents a conceptual shift: EVs are envisioned not simply as carriers of ligands or nucleic acids but as nanoscale platforms that facilitate high-density, spatially organized presentation of membrane-bound ligands. By clustering target cell receptors into signaling complexes, or “signalosomes”, this configuration can amplify the potency of EV-presented ligands by 10- to 1,000-fold compared to their soluble forms. However, this mechanism depends on direct physical contact between EV and target cell, inherently restricting the interaction to a one-EV-to-one-cell stoichiometry. Such a constraint may limit therapeutic scalability and tissue distribution, especially in complex *in vivo* environments.

**Figure 4:**
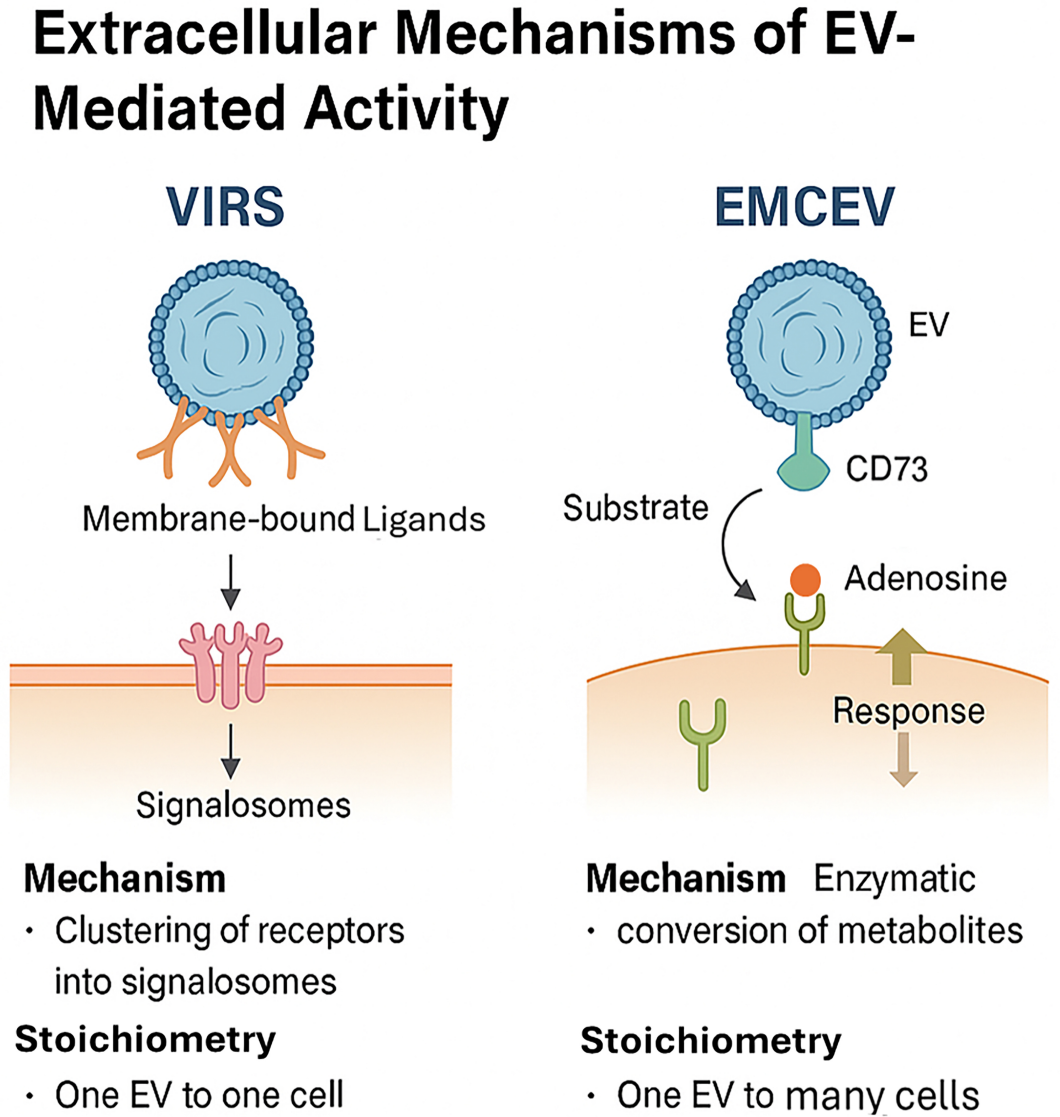
Comparison of the VIRS and EMCEV models, highlighting differences in mechanism and stoichiometry: (1) VIRS: Ligand clustering, signalosome formation, 1 EV : 1 cell; (2) EMCEV: Enzymatic conversion (e.g., CD73-mediated adenosine production), 1 EV : many cells. (Figure was created with AI-assisted technologies). VIRS: Vesicle-induced receptor signaling; EMCEV: enzymatic metabolite conversion by extracellular vesicles; EV: extracellular vesicle; CD73: ecto-5’-nucleotidase.

To address this limitation, Tan *et al*. proposed the External Modulation of Cell by EV (EMCEV) hypothesis^[36]^. This model posits that MSC-sEVs exert therapeutic effects through EV-associated enzymatic activity and microenvironmental modulation, rather than through vesicle internalization or receptor clustering alone. In the EMCEV framework, EVs act as extracellular catalytic agents - for example, via CD73-mediated conversion of extracellular adenosine monophosphate (AMP) to adenosine, which subsequently binds to purinergic receptors on nearby cells. Crucially, this mechanism allows a single EV to influence multiple surrounding cells, establishing a “one-to-many” stoichiometry and enabling broader paracrine effects without the need for EV uptake [Figure 4].

These extracellular models - particularly EMCEV - offer several advantages:

· Biological plausibility, grounded in known extracellular enzyme activity and receptor pharmacodynamics; · Improved pharmacokinetic alignment, consistent with observed dose-response relationships at low vesicle concentrations; · Direct linkage to therapeutic outcomes, through measurable surface protein activity or metabolite modulation.

Importantly, such mechanisms provide a rational basis for defining potency-related CQAs that do not depend on assumptions of intracellular delivery or gene silencing by miRNA cargo. As EV-based therapeutics advance toward clinical translation, mechanism-driven models such as VIRS and EMCEV will be pivotal in guiding potency assay development, regulatory classification, and product design.

### Critical reassessment of miRNAs as potency-associated CQAs beyond cargo delivery

#### Overrepresentation of miRNA research

Despite limited functional evidence, miRNAs have dominated EV research. A 2024 bibliometric survey reported that over 30% of EV-related publications focused on miRNA content, profiling, or putative functions^[37]^. This emphasis stems from:

· The perceived novelty and gene-regulatory potential of miRNAs; · The availability of high-throughput profiling tools [e.g., microarrays, quantitative polymerase chain reaction (qPCR), RNA sequencing (RNA-seq)]; · Ease of bioinformatic target prediction using seed region algorithms.

However, the prevalence of miRNA research has not translated into robust functional validation - particularly in the context of sEV-mediated delivery.

#### Misconceptions about miRNA enrichment

Multiple independent studies have shown that miRNAs represent only 2%-5% of small RNA cargo in MSC-sEVs^[38-41]^ compared to 19%-49% in most cells including MSCs^[38,42]^. This underrepresentation is often masked by normalization practices that compare miRNA abundance to non-EV molecules such as glycolytic enzyme glyceraldehyde-3-phosphate dehydrogenase (GAPDH) or total RNA input, resulting in misleading conclusions about enrichment. Direct comparisons of small RNA profiles in the above studies reveal that miRNAs are selectively loaded in EVs relative to their cellular source.

#### Low copy number: a functional barrier

Despite widespread claims that MSC-EVs exert their therapeutic effects via intracellular delivery of miRNAs, there remains no direct evidence that the quantity of miRNAs contained within EVs is sufficient to account for the observed increases in cellular miRNA levels following EV exposure.

Indeed, the biological plausibility of EV-mediated gene regulation via miRNAs is significantly challenged by the exceedingly low copy numbers of these RNAs within extracellular vesicles. Even among the most abundant miRNAs detected in EVs, quantitative analyses reveal copy numbers that fall several orders of magnitude below those required to elicit functional gene silencing in recipient cells. For instance:

· miR-133a-3p, a highly abundant miRNA in EVs derived from C2C12 myoblasts, was detected at a frequency of only 1 copy per 195^[43]^; · EBV-miR-BHRF1-2, a highly abundant viral miRNA, was present at 1 copy per 300 EVs from Epstein-Barr virus-transformed lymphoblastoid cell lines^[44]^; · Many of the most abundant miRNAs in EVs from plasma, prostate cancer plasma, dendritic cells, mast cells, seminal fluid, and ovarian cancer cells are present at 0.00001 to 0.1 copies per EV^[45]^.

In stark contrast, functionally active miRNAs within recipient cells typically exist at hundreds to thousands of copies per cell^[46-50]^, a concentration necessary to saturate the RNA-induced silencing complex (RISC) and mediate measurable post-transcriptional repression. For example, miR-122, a liver-enriched miRNA, is estimated to exist at ~120,000 copies per hepatocyte^[51]^.

Given this vast quantitative disparity, it is biologically implausible that the delivery of such miniscule miRNA doses via EVs - often below one copy per vesicle - could produce meaningful gene regulatory effects in recipient cells under physiological conditions. This strongly suggests that the reported elevation in cellular miRNA following EV treatment is more plausibly due to secondary regulatory effects rather than physical transfer of EV miRNAs. This raises serious doubts about the canonical RISC-dependent gene silencing model as the primary mechanism of EV-miRNA action, especially *in vivo*.

#### Incompatibility with RISC-mediated silencing

Canonical miRNA biogenesis involves Dicer processing of precursor stem-loop RNAs into mature miRNAs, which are then incorporated into Argonaute (AGO)-containing RISC to guide sequence-specific translational repression or, less commonly, mRNA cleavage. Proteomic analyses have shown that AGO proteins are either absent or present at extremely low levels in MSC-sEVs^[52]^. While precursor stem-loop RNAs have been reported to be enriched in MSC-EVs^[53]^, most miRNAs in MSC-EVs are mature, single-stranded forms^[38,39]^ and are not suited for RISC loading. Functional reporter assays have failed to show evidence of sEV-mediated gene silencing via canonical RNAi, even under optimized conditions with VSV-G engineered vesicles^[44]^.

#### Endosomal escape: a major bottleneck

Even when EVs are internalized, most are sequestered in endolysosomal compartments. Only a small fraction escapes into the cytosol, where RNAi machinery resides. Albanese *et al*. (2021) used a dual-luciferase reporter system to test whether single miRNA species delivered via EVs could mediate gene silencing^[44]^. Despite rigorous experimental controls and enhancements, no functional activity was detected - highlighting endosomal escape as a critical bottleneck.

### Summary for a miRNA-centric mechanism of action

**Figure 5:**
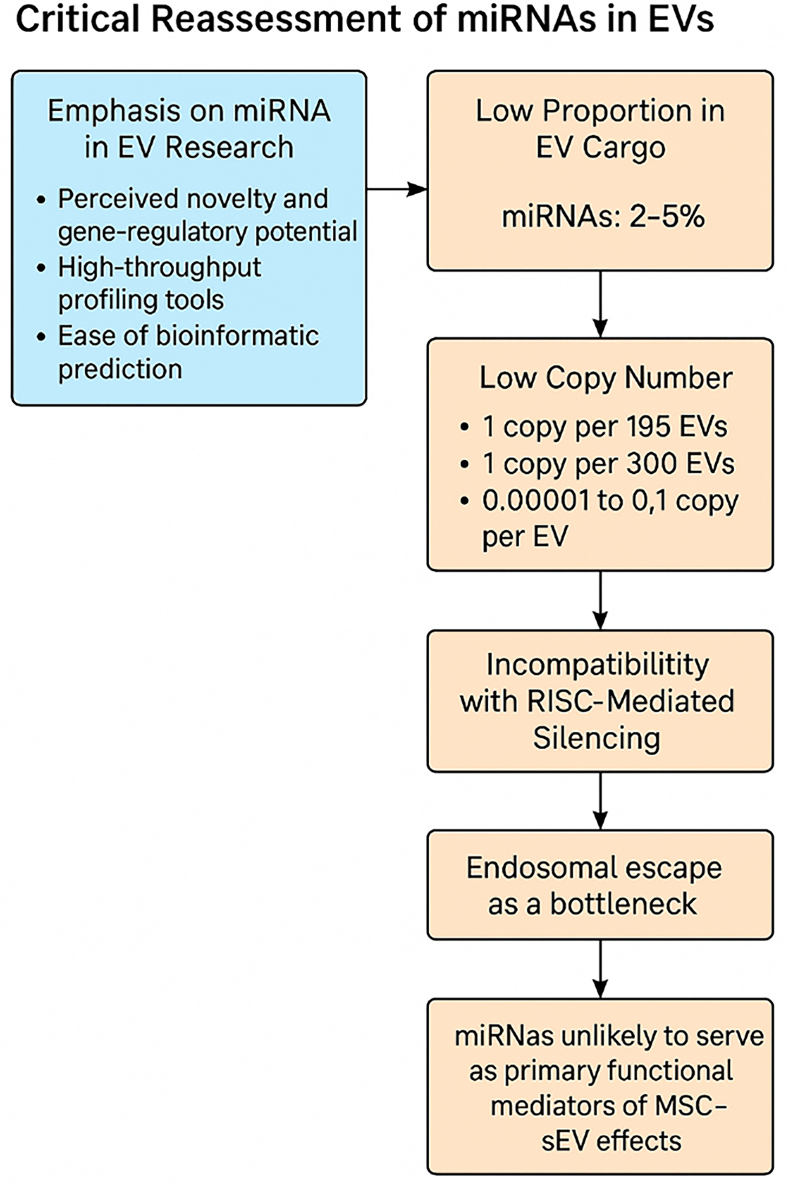
Critical data-driven assessment of a miRNA-centric mechanism of action (Figure was created with AI-assisted technologies). EVs: Extracellular vesicles; MSC: mesenchymal stem cell; sEV: small extracellular vesicle; miRNA: microRNA.

The cumulative evidence strongly suggests that miRNAs are unlikely to serve as primary functional mediators of MSC-sEV therapeutic effects. Their low abundance, inefficient delivery, and lack of RISC compatibility make them implausible drivers of the observed regenerative, anti-inflammatory, and immunomodulatory outcomes [Figure 5].

Although numerous studies report increases in cellular miRNA content following EV exposure, the quantitative insufficiency of miRNAs within EVs, coupled with the inefficient uptake of EVs by recipient cells, makes it unlikely that these increases result from direct delivery of miRNA cargo. Instead, current evidence more convincingly supports an indirect regulatory mechanism, whereby cellular miRNA elevation arises as a secondary response to EV-induced cellular signaling, rather than from the physical transfer of miRNAs from EVs to cells. Accordingly, while EV-associated miRNAs may serve as useful biomarkers of manufacturing consistency or reflect the physiological state of the parent MSCs, they should not be relied upon as functional potency CQAs. The development of robust potency assays should instead focus on mechanistically relevant and functionally predictive attributes, particularly those linked to direct extracellular or surface-mediated mechanisms of action, such as enzymatic activity or receptor-level modulation.

## CONCLUSION

While MSC-sEVs represent a transformative advancement in regenerative medicine, their path to clinical utility depends on the precise definition of identity and potency CQAs grounded in mechanistic reality. Among the proposed functional mediators, miRNAs have received disproportionate emphasis in literature and regulatory discourse. However, critical examination reveals that miRNAs are neither abundant nor reliably functional when delivered via native MSC-sEVs. Their low copy numbers, inefficient delivery, and lack of essential cofactors significantly limit their potential to act as meaningful therapeutic agents.

Instead of relying on miRNA-centric models, a shift toward extracellular or receptor-mediated mechanisms, such as ligand-receptor interactions or CD73-driven adenosine generation, may better reflect the true pharmacological action of MSC-sEVs. These mechanisms not only align with emerging evidence on sEV biodistribution and internalization dynamics but also offer more tractable endpoints for potency assay development. In this context, miRNAs may still serve as useful biomarkers of manufacturing consistency or cell state, but their utility as functional potency CQAs is highly questionable. Future development efforts should prioritize potency assays that reflect extracellular modes of action, integrate biodistribution and pharmacodynamic insights, and focus on protein- or activity-based metrics more directly tied to therapeutic outcomes. Reframing the discussion away from miRNAs and toward empirically grounded mechanisms will be essential for unlocking the full clinical potential of MSC-sEV-based therapies.

## DECLARATIONS

### AI and AI-assisted tools statement

AI and AI-assisted tools were used solely for grammar/spelling edits and for rendering assistance in figure preparation. The authors retain full responsibility for - and are the exclusive originators of - the manuscript’s concepts, structure, analyses, and conclusions.

### Copyright

© The Author(s) 2025.
